# Case Report: Interleukin-2 Receptor Common Gamma Chain Defect Presented as a Hyper-IgE Syndrome

**DOI:** 10.3389/fimmu.2021.696350

**Published:** 2021-06-24

**Authors:** Brahim Belaid, Lydia Lamara Mahammed, Aida Mohand Oussaid, Melanie Migaud, Yasmine Khadri, Jean Laurent Casanova, Anne Puel, Nafissa Ben Halla, Reda Djidjik

**Affiliations:** ^1^ Department of Medical Immunology, Beni-Messous University Hospital Center, Algiers, Algeria; ^2^ Faculty of Medicine, Benyoucef Benkhedda University of Algiers 1, Algiers, Algeria; ^3^ Department of Pediatrics A, Beni-Messous University Hospital Center, Algiers, Algeria; ^4^ Laboratory of Human Genetics of Infectious Diseases, Necker Hospital for Sick Children, INSERM UMR 1163, Paris, France; ^5^ Imagine Institute, University of Paris, Paris, France; ^6^ St Giles Laboratory of Human Genetics of Infectious Diseases, Rockefeller University, New York, NY, United States; ^7^ Howard Hughes Medical Institute, New York, NY, United States

**Keywords:** Interleukin-2 receptor gamma, combined immunodeficiency, hypomorphic mutations, hyper-IgE, inborn error of immunity

## Abstract

X-linked severe combined immunodeficiency (X-SCID) is caused by mutations of *IL2RG*, the gene encoding the interleukin common gamma chain (IL-2Rγ or γc) of cytokine receptors for interleukin (IL)-2, IL-4, IL-7, IL-9, IL-15, and IL-21. Hypomorphic mutations of *IL2RG* may cause combined immunodeficiencies with atypical clinical and immunological presentations. Here, we report a clinical, immunological, and functional characterization of a missense mutation in exon 1 (c.115G>A; p. Asp39Asn) of *IL2RG* in a 7-year-old boy. The patient suffered from recurrent sinopulmonary infections and refractory eczema. His total lymphocyte counts have remained normal despite skewed T cell subsets, with a pronounced serum IgE elevation. Surface expression of IL-2Rγ was reduced on his lymphocytes. Signal transducer and activator of transcription (STAT) phosphorylation in response to IL-2, IL-4, and IL-7 showed a partially preserved receptor function. T-cell proliferation in response to mitogens and anti-CD3/anti-CD28 monoclonal antibodies was significantly reduced. Further analysis revealed a decreased percentage of CD4^+^ T cells capable of secreting IFN-γ, but not IL-4 or IL-17. Studies on the functional consequences of IL-2Rγ variants are important to get more insight into the pathogenesis of atypical phenotypes which may lay the ground for novel therapeutic strategies.

## Introduction

X-linked severe combined immunodeficiency (X-SCID) is a life-threatening inborn error of immunity, accounts for approximately half of all cases of SCID (1–5). Most infants with SCID die within their first year of life in the absence of immune reconstitution *via* hematopoietic stem cell transplantation, due to severe and recurrent infections that begin in the first months of life, frequently associated with diarrhea and growth retardation. The infections may be viral, bacterial, and/or fungal, and vaccination with BCG may lead to disseminated infection (6).

X-SCID is caused by hemizygous pathogenic variants of the interleukin 2 receptor gamma (*IL2RG*) gene, organized in eight exons that encode the common γ chain, (IL-2Rγ or γc, also known as CD132), which is a part of the IL-2 high-affinity receptor and several interleukin receptors, including those for IL-4, IL-7, IL-9, IL-15 and IL-21 (7, 8). The γc protein is expressed on the surface of lymphoid, myeloid, and hematopoietic progenitor cells. The extracellular domain of the chain is encoded by exons 1–5, followed by a transmembrane domain encoded by exon 6, while the two last exons encode the intracellular portion which can cooperate with the Janus kinase family member 3 (JAK3) (9, 10), a signaling kinase that interacts with other JAK and STAT proteins in complex signal transduction through common gamma chain of cytokine receptor subfamily (11). These receptors’ engagement is crucial to lymphocyte activation, proliferation, and function. Impaired signaling downstream of IL-7 (12) and IL-21 (13–17) explains, at least in part, the absence of T cells and impaired B-cell function, respectively, of X-SCID patients. Altogether, the absence of normal IL-4, IL-7, IL-9, IL-15, and IL-21 signaling leads to the almost complete absence of T and NK cells, with nonfunctional B cells observed in the typical T^-^B^+^NK^-^ X-SCID patients carrying amorphic mutations of *IL2RG* (4, 6). On the other hand, atypical cases of X-SCID have been described in male patients carrying hypomorphic mutations of *IL2RG*, resulting in either a residual expression of γc protein with a weaker affinity for IL-2, or a weaker interaction of γc with the downstream kinase JAK3 leading to an impaired signaling (18–21).

The pathogenic genetic variations causing X-SCID are found throughout the *IL2RG* sequence, with missense mutations being the most common ones, followed by nonsense mutations and insertions/deletions (7). Mutations resulting in a complete absence of IL-2Rγ expression are likely to result in the classical X-SCID phenotype. On the other hand, missense mutations in the IL2RG locus have been mainly associated with less severe phenotypes referred to as “leaky or atypical X-SCID” (22–24). Furthermore, some atypical X-SCID cases with hypomorphic mutations may display, later in life, chronic lung diseases, warts, and recurrent respiratory and gastrointestinal tract infections, as well as other atypical clinical manifestations (25).

We report the case of a male child with an atypical clinical presentation carrying to a missense mutation of *IL2RG* gene, with normal T, B, and NK cell counts, high serum IgE levels, persistent eczema, and recurrent sinopulmonary infections. We performed a deep immunophenotyping and functional tests to better characterize the impact of this *IL2RG* variation. This report adds to the ever-growing knowledge on atypical X-SCID disorders and contributes to a better understanding of the clinical variability associated with the *IL2RG* gene defect.

## Results

### Case Presentation

**Figure 1:**
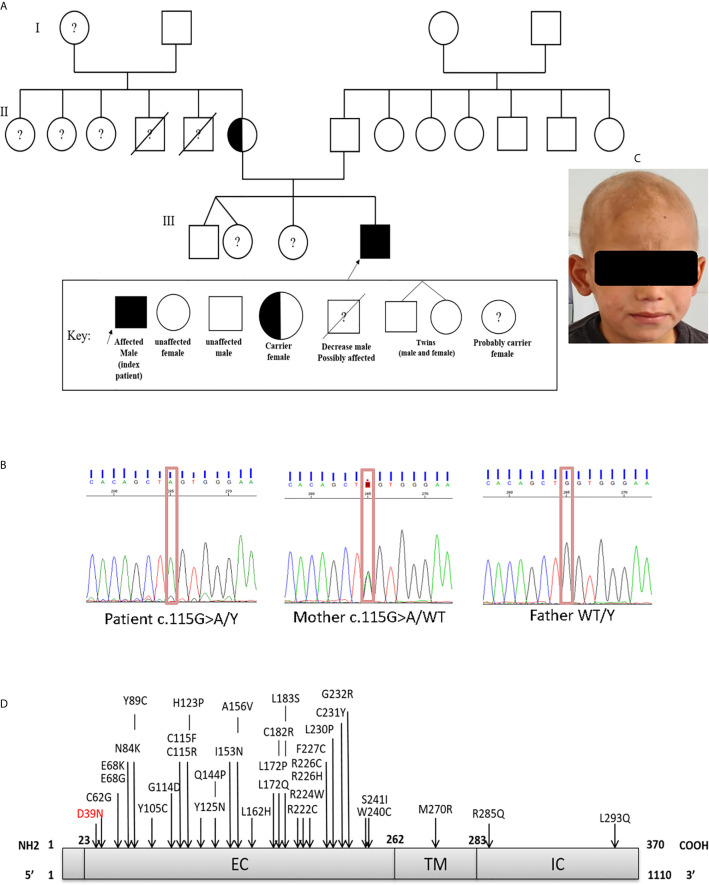
**(A)** Family pedigree of the patient. **(B)** Sanger sequencing confirmed the presence of the mutation in exon 1 (c.115G>A; p.Asp39Asn). The heterozygous (mother) and WT states. **(C)** Scarring alopecia, hypotrichosis with ringworm of the scalp. **(D)** Schematic diagram of the IL-2RG protein, with its extracellular (EC), transmembrane (TM), intracellular (IC) domains. In red the mutation carried by the patient and in black the positions affected by the mutations already described in the literature.

The patient, a 7-year-old Algerian male born to unrelated healthy parents, was admitted to the department of pediatrics at Beni Messous University Hospital due to recurrent sinopulmonary infections and treatment-resistant eczema. He was born at term by cesarean section with a birth weight of 3400g and received all the routine vaccinations with no noticeable complications. Besides moderate growth retardation, his mental and psychomotor development was normal. Recurrent upper and lower respiratory tract infections began at four years of age, requiring antibiotic therapy and hospitalization. In addition, the patient had several episodes of ear, nose, and throat (ENT) infections with a marked increase of C-reactive protein (CRP) concentration. He suffered from severe eczema since the age of 4 years, refractory to topical corticosteroids; one year later, he also developed a cutaneous leishmaniasis treated successfully with pentavalent antimonial salts (meglumine antimoniate) by parenteral administration. Investigation of his family history (**Figure 1A**) revealed that two of his maternal uncles displayed similar clinical manifestations, with one of them who has been treated for a celiac disease and who died of infectious complications at 22 years of age, while the other one, who was treated for bronchiectasis and severe refractory eczema, died at the age 28 years.

The patient who was underweight exhibited during the first physical examination, at the age of 6 years, a ringworm of the scalp accompanied by hair loss (**Figure 1C**), and an eczema present on his ears, elbow folds, and bursae. His first blood test (**Table 1**) showed a high white blood cell (WBC) count 13800/mm3 (neutrophils 57%, lymphocytes 21%, monocytes 6.5%, eosinophils 15.5%), and normal liver and kidney function tests. Measurement of serum immunoglobulin concentrations showed a pan-hypergammaglobulinemia: IgG 1755 mg/dL (normal or elevated IgG subclasses), IgA 219 mg/dL, IgM 145 mg/dL, and IgE 31120 IU/mL. A flow cytometry analysis of the different lymphocyte subsets (**Supplementary Figure S1**) revealed normal numbers and frequencies of CD3^+^ T cells 84% (absolute count 2434/mm^3^), CD3^+^CD8^+^ T cells 61.5% (1782/mm^3^), CD19^+^ B cells 9.5% (275/mm^3^), and CD56^+^NK cells 6.5% (188/mm^3^). However, the patient had low numbers of CD3+CD4+ T cells 15.5% (449/mm^3^), with inverted CD4^+^/CD8^+^ ratio (0.25).

**Table 1:** Immunologic characteristics of the patient with hypomorphic/atypical X-SCID.

Parameters	Result	Normal range
Complete blood count		
White blood cells (cells/µl)	*13800*	4400 – 9500
Lymphocytes (cells/µl)	2898	1900 – 3700
Neutrophils (cells/µ)	*7866*	2600 – 6300
Eosinophils (cells/µl)	*2139*	0 – 200
monocytes (cells/µl)	897	300 – 900
Hemoglobin (g/dl)	12.5	12 – 17
Platelet (×10^3^ cells/µl)	*489*	150 – 450
Immunoglobulin levels		
IgG (mg/dl)	*1755*	680 – 1180
IgG_1_ (mg/dl)	*1312.4*	288 – 918
IgG_2_ (mg/dl)	144	44 – 375
IgG_3_ (mg/dl)	*160.1*	15.5 – 85.3
IgG_4_ (mg/dl)	44.6	0.4 – 99.2
IgA (mg/dl)	*219*	70 – 190
IgM (mg/dl)	*145*	32 – 98
IgE (IU/ml)	*31120*	<45
Lymphocyte subsets		
CD3^+^ T cells/µl	2434	1200 – 2600
αβT cells (TCRαβ+TCRγδ−/CD3+)/T cells%	*83*	85 – 95
γδT cells (TCRαβ−TCRγδ+/CD3+)/T cells%	17	7 – 20
CD4^+^ T cells/µl	*449*	650 – 1500
CD4_memory_CD45RO^+^ T cells/CD4^+^ T cells%	*65.5*	13 – 30
CD4_Naive_CD45RA^+^CCR7^+^ T cells/CD4^+^ T cells%	20.13	15.5 – 59.4
CD4_CM_CD45RA^-^CCR7^+^ T cells/CD4^+^ T cells%	19.32	12.2 – 26.2
CD4_EM_CD45RA^-^CCR7^-^ T cells/CD4^+^ T cells%	*54.31*	10.6 – 34.2
CD4_TEMRA_CD45RA^+^CCR7^-^ T cells/CD4^+^ T cells%	6.24	4.5-43.6
CD4_RTE_CD45RA^+^CD31^+^/CD4^+^ T cells%	24.5	19.4 – 60.9
CD8^+^ T cells/µl	*1782*	370 – 1100
CD8_memory_CD45RO^+^ T cells/CD8^+^ T cells%	*82*	8 – 37
CD8_Naive_CD45RA^+^CCR7^+^ T cells/CD8^+^ T cells%	*1.6*	5.5 – 39.7
CD8_CM_CD45RA^-^CCR7^+^ T cells/CD8^+^ T cells%	*0.25*	1.2 – 3.8
CD8_EM_CD45RA^-^CCR7^-^ T cells/CD8^+^ T cells%	*91.98*	20.1– 44.7
CD8_TEMRA_CD45RA^+^CCR7^-^ T cells/CD8^+^ T cells%	*6.17*	21.5 – 61
CD4^+^/CD8^+^ Ratio	*0.25*	1.5 – 2.9
DN T cells (CD4−CD8−/CD3+TCRαβ+)	0.35	0.18 – 2.81
Regulatory T cells (CD25+IL7R−/CD3+CD4+)	11.52	4 – 14
CD19^+^B cells/µl	275	270 – 860
B_naive_ (CD27^-^IgD^+^)/CD19^+^ B cells %	*68.11*	69.4 – 80.4
B_switched memory_ (CD27^+^IgD^-^)/CD19^+^ B cells %	8.09	5.2 – 12.1
B_unswitched memory_ (CD27^+^IgD^+^)/CD19^+^ B cells %	*7.13*	7.5 – 12.4
B_transitional_ (CD24^++^CD38^++^)/CD19^+^ B cells %	*4.3*	4.5 – 9.2
B_plasmablast_ (CD24^-^CD38^++^)/CD19^+^ B cells %	2.43	0.7 – 3.5
B_CD21(-/low)_ (CD21^low^CD38^low^)/CD19^+^ B cells %	*17.5*	0.9 – 3.5
CD3-CD16+CD56^+^ NK cells/µl	188	100 – 480

Values in boldface and italics are abnormal.

CM, Central memory; EM, effector memory; TEMRA, Terminally differentiated T cells; RTE, Recent Thymic Emigrant, DN, Double negative.

Further characterization of the lymphocyte subpopulations (**Table 1**) showed a substantial shift towards a memory phenotype (**Supplementary Figure S2**) with an increased percentage of CD45RO+ CD4+ and CD45RO+ CD8+T cells, low percentages of naïve T cells (CCR7+CD45RA+), and high percentages of CD4+ and CD8+ effector memory T cells (TEM) (CCR7-CD45RA-) (**Supplementary Figure S3**). By contrast, the frequency of CD4^+^ T cells displaying the phenotypical characteristics of recent thymic emigrants (RTE) (CD4^+^CD45RA^+^CD31^+^) was in the normal range (**Supplementary Figure S4**). similarly, the evaluation of peripheral B lymphocytes demonstrated a normal distribution of B cell subsets (**Supplementary Figure S5**). In fact, despite an expansion of CD19^+^CD21^–/low^ B cell subset, the Percentages of naïve B cells (CD19^+^IgD^+^CD27^-^), switched and unswitched memory B cells (CD19^-^CD27^+^IgD^-^ and CD19^+^CD27^+^IgD^+^, respectively), transitional B cells (CD19^+^CD24^++^CD38^++^), as well as plasmablasts (CD19^+^CD24^-^CD38^++^) did not significantly differ from values observed in healthy age-matched individuals.

**Figure 2:**
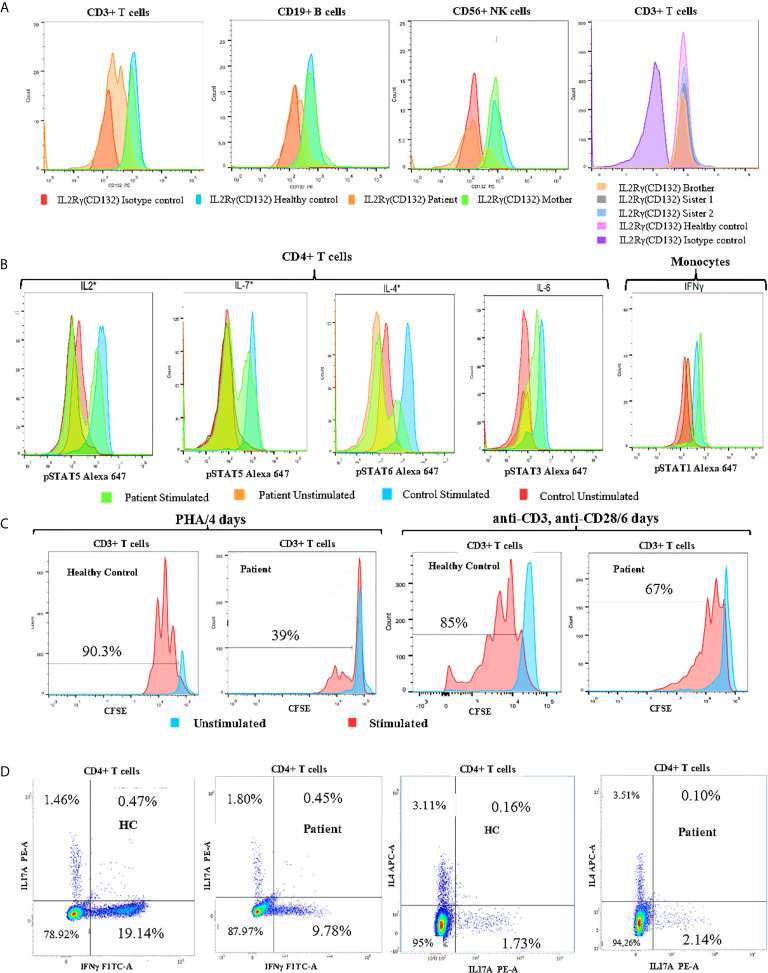
Immune functional and proliferation assays in subsets of lymphocytes. **(A)** Flow cytometry analysis of CD132 (γc) expression on T, B and NK cells of the patient, his mother, and healthy control, and screening of siblings for CD132 expression on T cells. **(B)** Histogram of CD4+ T cell and monocyte response with STAT phosphorylation to several γc dependent (IL-2, IL-4, IL-7) and independent (IL-6, IFN-γ) cytokines in whole blood of patient. **(C)** Analysis of proliferation of healthy control (HC) and patient CFSE labeled cells with the indicated stimuli: 4 days of culture with Phytohemaglutinin (PHA) and 6 days with anti-CD3/anti-CD28 monoclonal antibodies. **(D)** The percentages of IL-17A, IFNγ and IL-4-producing CD4+ T cells from patient and age-matched control using PMA/Ionomycin stimulation in the presence of monensin for 5 hours. *Gamma chain dependent cytokines; the others are gamma chain independent.

Given the presence of refractory eczema associated with high serum IgE concentrations, hypereosinophilia, and recurrent bacterial infections, we initially suspected a hyper-IgE syndrome. Therefore, we used a scoring system which the NIH has developed to help in the diagnosis of these patients based on both the immunologic and clinical features of the syndrome. Indeed, a score of ˃40 points makes the diagnosis of AD-HIES highly probable and unlikely with a score of < 20 points (26). When the scoring criteria were applied to our patient, the overall clinical and immunological presentation was between STAT3-HIES and DOCK8-CID with 39 points in the NIH-HIES score. Therefore, IL-6-induced expression of intracellular phospho-STAT3 (pSTAT3) was determined in CD4+T-cells by whole blood flow cytometric analysis, which included assessment of surface IL-6RA and gp130 expression. No difference for pSTAT3 expression was detected between patient and healthy control (**Figure 2B**).

In order to identify the molecular defect behind the pathology, whole-exome sequencing (WES) identified the presence of a hemizygous missense mutation (c.115G>A) in exon 1 of the *IL2RG* gene. The presence of the missense mutation was further confirmed by Sanger sequencing (**Figure 1B**). This substitution resulted in an amino acid change from Aspartic Acid to Asparagine at position 39 (p.Asp39Asn) of the mature protein. Bioinformatic analysis predicted that the p.Asp39Asn mutation was predicted to be probably pathogenic and affected a highly conserved amino-acid residue (SIFT-deleterious; PolyPhen2-possibly damaging), (**Supplementary Figure S6**). The high CADD score of 24.2 (sensitivity: 0.41; specificity: 0.98) also suggested a probably damaging effect of the variant. No additional mutations were found in any other known disease-causing genes (**Supplementary Figure S7**).

We also found that the patient’s mother was a heterozygous carrier of this variant. An immunostaining with anti-CD132 and flow cytometry analysis showed a significant reduction of γc surface expressions on T, B and NK cells of the patient when compared to his parents, siblings, or healthy control (**Figure 2A**).

In order to study the impact of Asp39Asn amino acid substitution on immune cell signaling, we measured STAT phosphorylation in CD4+ T cells and monocytes. The mutation was associated with defective phosphorylation of STAT5, STAT6, and STAT5 upon the stimulation of CD4+ T cell with IL-2, IL-4, and IL7, respectively. However, γc independent signaling in monocytes (IFN-γ induced pSTAT1) was not affected (**Figure 2B**). Evaluation of CD3+ T cell proliferation through carboxyfluorescein succinimidyl ester (CFSE) staining revealed a significant defect of mitogen phytohemagglutinin (PHA)-induced cell proliferation compared to healthy control (39% *vs.* 90.3%). Similarly, the activation of CD3+ T cells of the patient with anti-CD3 and anti-CD28-coated beads led to a reduced proliferation compared to healthy control (67% *vs.* 85%) (**Figure 2C**). Taken together, these data suggest that the patient has a “leaky” form of SCID.

To better characterize the immunologic defects associated with the patient’s mutation, the percentages of CD4+IFNγ+ (Th1), CD4+IL4+ (Th2), and CD4+IL-17A+ (Th17) cells were assessed in PBMCs by flow cytometry. The patient exhibited a significant reduction in the proportion of IFNγ producing CD4+ T cells (Th1) and normal CD4+IL4+(Th2) and CD4+IL17A+(Th17) frequencies when compared to an age-matched control subject (**Figure 2D**). CD4+CD25^high^CD127^low/-^FoxP3+ Regulatory T-cells represent 11,52% of CD4+ T-cells. Based on these findings, we have recommended allogeneic hematopoietic stem cell transplantation as a definitive treatment. Since, the patient regularly performed immunoglobulin replacement therapy and received prophylactic antimicrobial (co-trimoxazole, sulfamethoxazole, and itraconazole). He is checked for signs of malignant disease on a regular basis, but so far, no lymphoma or other tumors have been noted.

## Discussion

This report describes the case of a seven-year-old Algerian patient carrying a hemizygous missense (c.115G>A) variant of *IL2RG* gene, and results in replacement of aspartic acid by asparagine at position 39 (p.Asp39Asn) in the extracellular domain of the protein (**Figure 1D**). As far as we are aware, only a single other case carrying a same mutation has been reported in the literature (27, 28).

Our patient displayed unusual clinical and immunological features with persistent eczema and ringworm of the scalp, associated with recurrent sinopulmonary infections, high IgE levels, and peripheral blood eosinophilia, which initially suggested a hyper-IgE syndrome. A reported case (27, 28) with the same amino acid substitution and a more severe clinical phenotype (**Table 2**) was mainly characterized by repetitive infections and protracted diarrhea starting around nine months of age without a history of eczema. Given the different clinical and immunological manifestations, it seems reasonable to hypothesize that other factors, such as epigenetic, environmental, and ethnic contributions, could affect the disease’s evolution (29). These findings emphasize the issues that might be involved in the relationship between the environment and the genome in multifactorial disorders, in which numerous environmental factors are included (30). As reported by Dworkin et al, attempting to understand the molecular interaction of genetic background effects using model organisms, found that were partly associated with differentially expressed genes where quantitative transcription level differences correlated with variation for the phenotype (31). Furthermore, Lachance et al. studied genetic background effects on an X-linked gene in Drosophila melanogaster leading to wing defects. The natural variant was placed into multiple backgrounds, then they assessed penetrance and expressivity of wing defects. They found significant complex interactions that were affected by the genetic background (32).

**Table 2:** Characteristics of reported patient carrying the IL2RG^115G>A^ missense mutation.

	DiSanto et al. (16), de saint basile et al. (17)	Belaid et al.
**Patient (age, ethnicity)**	5 1/2-year-old Portuguese male	7-year-old Algerian male
**Consanguinity**	No	No
**Medical history**	protracted diarrhea, otitis media	Persistent eczema, recurrent infections, cutaneous leishmaniasis
**Family history**	3 maternal uncles and 2 older brothers died of bronchopneumonitis with bronchiectasis, skin infections, protracted diarrhea, and failure to thrive	2 maternal uncles died of recurrent infections with bronchiectasis.
**Onset**	9-month-old	4-year-old
**Immunological findings**		
**Thymic shadow**	extreme thymic atrophy with rare Hassal’s corpuscles	n.a
**Blood count**	Normal	Normal
**Immunophenotype**	T^low^, B^+^, NK^+^	T^+^, B^+^, NK^+^
**γδ T cells**	Normal	Normal
**Microbiological examination**	Poliovirus, Bordetella pertussis	leishmania infantum
**Extended immunophenotyping**	T cells skewed to the memory phenotype	T cells skewed to the memory phenotype, profound decreased of naïve T cells, normal distribution of B cells, reduced Th1.
**Immunoglobulin levels**	Normal with elevated IgE levels	Hypergammaglobulinemia with extremely high IgE levels.
**TCR-Vβ repertoire**	limited TCRβ heterogeneity and diminished functional activity	n.a
**Lymphocyte proliferation**	Reduced	Reduced
**IL2RG expression**	n.a	Severely reduced (by FCM)
**IL21/STAT3 phosphorylation**	n.a	n.a
**IL2 &IL7/STAT5 phosphorylation**	n.a	Partially defective
**IL4/STAT6 phosphorylation**	n.a	Partially defective
**TRECs**	n.a	n.a

n.a, not assessed; FCM, flow cytometry; TRECs, T-cell receptor excision circles.

Both Disanto et al, and de Saint Basile et al. had reported the same case.

Typically, patients with genetic mutations of the *IL2RG* gene are classically characterized by the absence or severe reduction of T and NK cell numbers, as well as the presence of non-functional B cells (33), these mutations are mainly localized within the exons 3-5 (34, 35). Missense mutations are the most common pathogenic changes observed, followed by nonsense variants and insertions/deletions (35). On the other hand, atypical clinical presentations of X-SCID have been also described in patients carrying missense mutations of *IL2RG* resulting in the expression of lower amounts of γc protein with conserved binding affinity for IL-2, or in its reduced interaction with JAK3, and thus impairing T cell activation (9, 36). However, such mutation requires assessing a cell line from the affected patient to evaluate γc cell surface expression and/or IL2RG mRNA transcripts as a direct proof if the mutation is deleterious. In the present case, an accurate diagnosis of atypical X-SCID was initially compromised because of the unusual clinical presentation, with almost normal development and growth, a low number of infections during the first 3 years of life. Moreover, the most first-line immunological investigations showed normal percentages and numbers of total T, NK, and B cells, but CD4+ T cell counts decreased while CD8+ T cell counts were expanded, combined with a polyclonal hypergammaglobulinemia; hence, these findings were initially inconsistent with X-SCID. Atypical cases with normal numbers of T and NK cells are very unusual and were previously reported in only few cases with *IL2RG* mutations (37–41), and some *IL2RG* mutations can lead to functional lymphocyte abnormalities rather than cell development defects, as some patients with normal lymphocyte differentiation and normal thymus biopsies were reported (37). Such phenotype can be a direct consequence of a residual γc expression providing sufficient signal for normal T and NK cell development. This is in accordance with the preserved signaling by the IL-7R and IL-15R, as it has been shown before by Smyth et al. that signal transduction by the IL-7R is crucial for T-cell development but is dispensable for NK cell development, whereas adequate signaling *via* the IL-15R is essential for NK cell but not for T-cell development (42, 43). This mechanism corroborates with our findings, suggesting that the amount of γc required for the correct signaling of various signaling pathways is different.

In addition to the aforementioned findings, the T lymphocyte differentiation and maturation skewed toward memory phenotype (CD45RO^+^), combined with increased counts of total CD8^+^ T cells as well as expansion of CD45^-^CCR7^-^ effector memory for both CD4^+^ and CD8^+^ T cells. This condition probably is in consistence with persistent viral infection (44), although no virological confirmation was possible. On other hand, thymic stromal lymphopoietin (TSLP) is another cytokine that is not a member of the γc family but has overlapping functions with IL-7 (45). Indeed, whereas the IL-7 receptor contains IL-7Rα and γc, the TSLP receptor consists of IL-7Rα and TSLPR, which is closely related to γc (46, 47). *IL2RG* ^−/−^ mice treated with recombinant TSLP, which cannot respond to IL-7 or other γc family cytokines, lead to a partial increase in CD8+ T cell numbers (48). Moreover, TSLP promotes the survival of CD8+ T cells in both normal and lymphogenic conditions (49).

In addition, our patient displayed moderately reduced capacity of T cells to proliferate in response to PHA or anti-CD3/anti-CD28 stimulation, reflecting disturbed IL-2 signaling. Indeed, IL-2 is key growth factor required for T cell expansion and promotes the proliferation and survival of activated T cells (50). Thus, it influences effector T cell differentiation and promotes fate decisions in activated T cells (51).

Furthermore, our patient had normal percentages of switched memory B cells, transitional B cells, and plasmablasts, but negligible reduction of naïve B cells, and unswitched memory B cells. There may exist a threshold level of γc expression necessary for normal B cell function, consequently, the collaborative IL-4 and IL-21 signaling might be sufficient for humoral responses (52–55). In contrast, we found signs of misguided enhanced B cell activity reflected by a large CD21^low^CD38^low^ B cell population, in addition to a polyclonal hypergammaglobulinemia. CD21^low^CD38^low^ B cells are a distinct B cell population that is mainly associated with manifestations of chronic immune activation, lymphoproliferation, and autoimmunity (56–58). Interestingly, in our case there was no evidence of conditions considered to be autoimmune or lymphoproliferation. This is in consistence with normal numbers of regulatory T cells (T reg) in indexed patient, although IL-2 is also critical for the development of Tregs in the thymus and for their maintenance and function in the periphery (59). In addition, high IgG1 and IgG3 levels were also observed which can recognize protein and viral antigens (60, 61). In fact, viral infections in general lead to IgG antibodies of the IgG1 and IgG3 subclasses, with IgG3 antibodies appearing first in the course of the infection (60, 61).

In the case reported by DiSanto et al., with the same mutation as the present patient, a reduced capacity to splice a correct-sized transcript, leading to the production of a nonfunctional transcript containing an insertion of 27 bp, and a reduced amount of a normal sized transcript containing a single amino acid substitution has been shown (28). Thus, they demonstrated that splicing of exons 1 and 2 normally generates the codon GAT, but with the base change, the resultant codon becomes AAT, and the point mutation (D39N) appeared not to impair IL-2 binding or its subsequent endocytosis (28). Therefore, a residual expression of *IL2RG* transcripts with normal length may account for the limited expression of γc detectable at the cell surface. Unfortunately, the size of the transcripts and γc protein were not assessed in the current work. However, our data showed that γc expression and STAT5/STAT6 phosphorylation were reduced but not completely abolished. These findings are consistent with a moderate phenotype suggesting that this mutation (p.Asp39Asn) is hypomorphic.

Interestingly, our patient developed atopic dermatitis-like skin lesions and alopecia associated to IgE hyperproduction (31,120 IU/ml) and hypereosinophilia (2139 cells/µl), such conditions were surprisingly rare in reviewed patients carrying *IL2RG* hypomorphic mutations. Indeed, only four patients, suffering from eczema or other skin rashes, were reported in the literature (15, 62–64). Milner et al. demonstrated that reduced T-cell activation caused by a weak signaling has been associated with a skew towards the development of T helper type 2 cells (Th2 cells) (62), this may suggest its profile as a default differentiation pathway partly underlying the features of skin lesions in these patients. This is a typical finding in patients with Omenn syndrome in whom expanded T-cell clones were consistently found to be predominantly of TH2 type (65), and to secrete IL-4/IL-13, IL-5 and IL-9, which promote immunoglobulin class-switching to IgE, activates eosinophils, and activates mast cells, respectively (66–69). These results are consistent with our patient, who showed normal percentage of IL-4 producing T cells and low proportions of IFN-γ producing T cells compared to age-matched control subject (**Figure 2D**), suggesting that Th2-type cytokines are central in the pathogenesis of this hyper-IgE production. Moreover, previous studies have also demonstrated that B cells from X-SCID patients with decreased expression of γc can respond to IL-4 *via* a type II IL-4R complex composed of IL-4Rα/IL-13R chains (70–72).

In conclusion, X-SCID is an inborn error of immunity that manifests as different clinical phenotypes, from milder to severe disease. Most of the attention focused on the possible relationship between mutations of the *IL2RG* gene and clinical/immunological features. Further investigations are required to achieve a better classification of the disease. We believe that unusual clinical and laboratory observations may be very useful to unravel complex diseases and help find novel gene-function relationship laying the ground for novel targeted therapeutic approaches.

## Data Availability Statement

The original contributions presented in the study are included in the article/**Supplementary Material**. Further inquiries can be directed to the corresponding author.

## Ethics Statement

The studies involving human participants were reviewed and approved by Comité d’éthique du CHU Beni Messous. Written informed consent to participate in this study was provided by the participants’ legal guardian/next of kin. Written informed consent was obtained from the minor(s)’ legal guardian/next of kin for the publication of any potentially identifiable images or data included in this article.

## Author Contributions

BB drafted the manuscript and contributed in collecting and analyzing the data. LM designed and performed experiments, analyzed the data, and reviewed the manuscript critically. AM, YK, and NB, cared for the patient and provided clinical data. AP, MM, and JC reviewed the manuscript critically and suggested changes to the final version and did the genetic testing. RD reviewed the manuscript and conceptualized the paper. All authors contributed to the article and approved the submitted version.

## Funding

The French National Research Agency (ANR) under the “Investments for the Future” program (ANR-10-IAHU-01), ANR-FNS LTh-MSMD-CMCD (ANR-18-CE93-0008-01), The Rockefeller University and the National Institutes of Health (# R01AI127564).

## Conflict of Interest

The authors declare that the research was conducted in the absence of any commercial or financial relationships that could be construed as a potential conflict of interest.

## Abbreviations

FOXP3, forkhead box P3; HIES, Hyper-IgE syndrome; IFN, Interferon; IL, Interleukin; IL2RG, Interleukin 2 receptor gamma; JAK3, Janus kinase 3; γC, common gamma chain; NK, Natural Killer; PBMC, Peripheral Blood Mononuclear cells; PHA, phytohemagglutinin; PMA, Phorbol 12-myristate 13-acetate; STAT, Signal transducer and activator of transcription.; SCID, Severe combined immunodeficiency; Th, Helper T lymphocyte.
