# Supplementary figures and images for: Case Report: Interleukin-2 Receptor Common Gamma Chain Defect Presented as a Hyper-IgE Syndrome
Source: Front Immunol. 2021 Jun 24;12:696350. doi: 10.3389/fimmu.2021.696350 (PMC8264782; doi:10.3389/fimmu.2021.696350)

**S1**


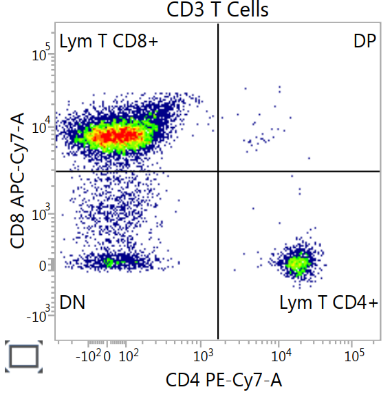

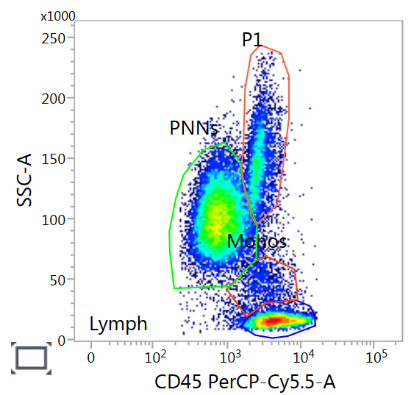

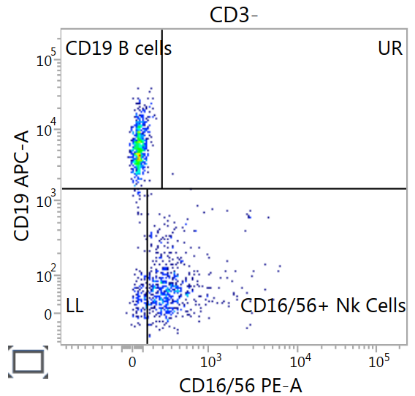


**
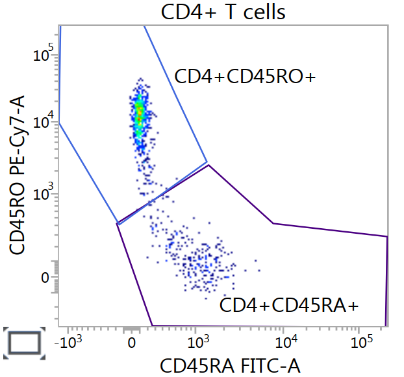
S2**


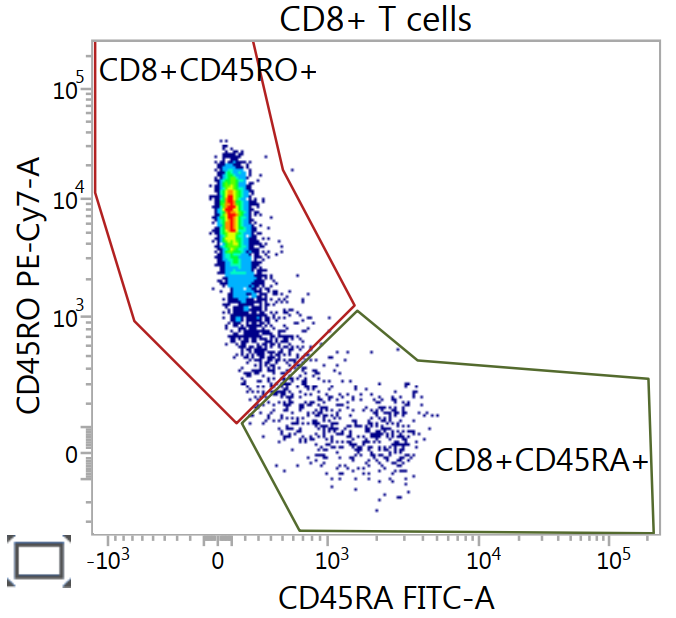


**
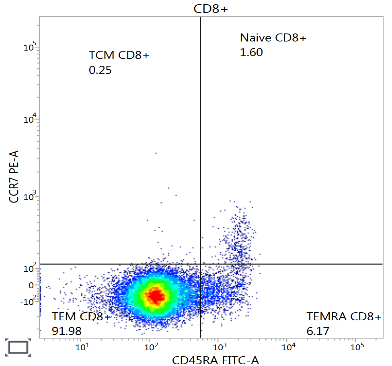

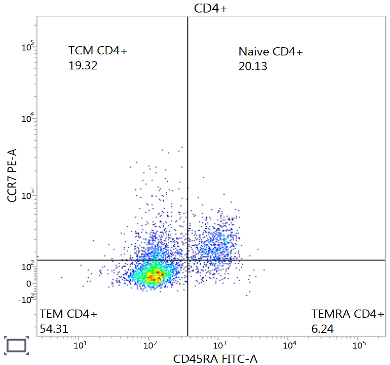
S3**

**S4**


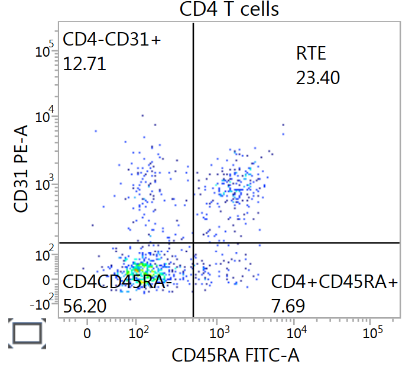


**
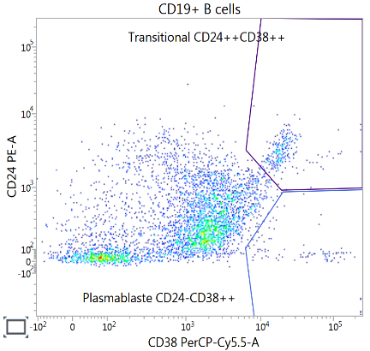

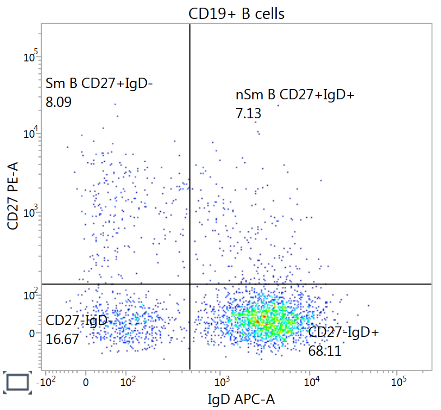
S5**

**
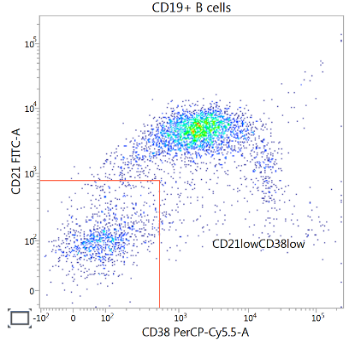
**

**
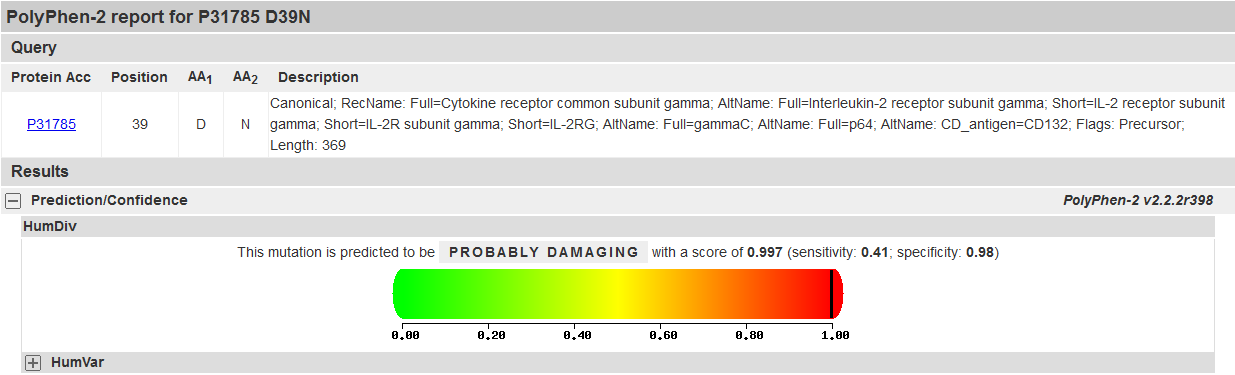
S6**

**
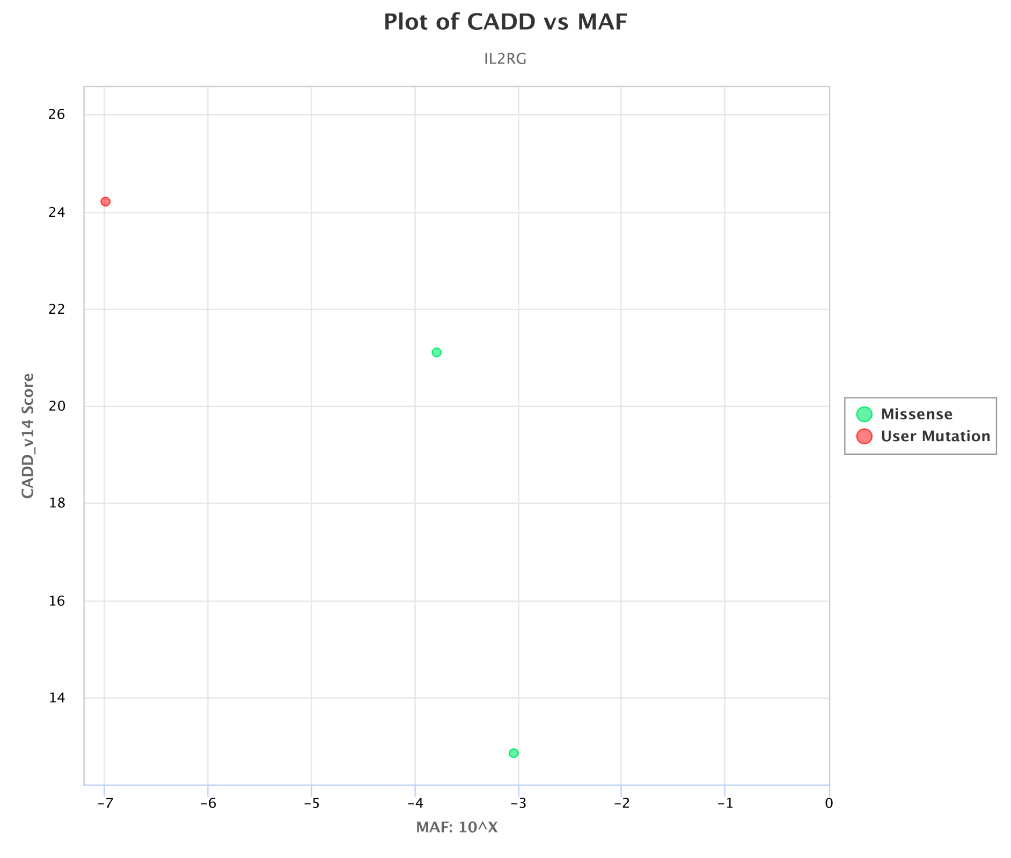
S7**

All variants reported in GnomAD

Only at hemizygous or homozygous state

Supplement: Supplementary file 1 [file DataSheet_1.docx]
